# Peptide-Based Hydrogels and Nanogels Containing Gd(III) Complexes as *T*_1_ Relaxation Agents

**DOI:** 10.3390/ph15121572

**Published:** 2022-12-16

**Authors:** Elisabetta Rosa, Fabio Carniato, Lorenzo Tei, Carlo Diaferia, Giancarlo Morelli, Mauro Botta, Antonella Accardo

**Affiliations:** 1 Department of Pharmacy, Research Centre on Bioactive Peptides (CIRPeB), University of Naples “Federico II”, Via Montesano 49, 80131 Naples, Italy; 2 Department of Science and Technological Innovation, University of Piemonte Orientale “A. Avogadro”, Viale T. Michel 11, 15121 Alessandria, Italy

**Keywords:** peptide hydrogels, nanogels, supramolecular assembly, MRI, contrast agents, diagnosis

## Abstract

New peptide-based hydrogels incorporating Gd(III) chelates with different hydration states, molecular structures and overall negative charges ([Gd(BOPTA)]^2−^), [Gd(DTPA)]^2−^, and ([Gd(AAZTA)]^−^) were prepared and characterized. N-terminal Fmoc- or acetyl-derivatized hexapeptides (K1, K2 and K3) containing five aliphatic amino acids (differently ordered Gly, Ala, Val, Leu and Ile) and a charged lysine at the amidated C-terminal were used for the formation of the hydrogels. Particular attention was paid to the investigation of the morphological and rheological properties of the nanoparticles, in addition to the assessment of the ability (relaxivity) of the confined complexes to accelerate the longitudinal relaxation rate of the water protons localized in the polymeric network. The relaxivity values at high magnetic fields (>0.5 T) of the paramagnetic hydrogels appear to be more than five times higher than those of isolated chelates in an aqueous solution, reaching a value of 25 mmol^−1^ s^−1^ for Fmoc-K2+[Gd(BOPTA)]^2−^ at 0.5 T and 310 K. Furthermore, an interesting trend of decrease of relaxivity with increasing the degree of rigidity of the hydrogel was observed. The type of interactions between the various complexes and the polymeric network also plays a key role in influencing the relaxivity values of the final materials. Nanogels were also obtained from the submicronization of the hydrogel containing [Gd(BOPTA)]^2−^ chelate. Circular dichroism, dynamic light scattering and relaxometric investigations on these nanoparticles revealed the formation of nanogels endowed with higher relaxivities (*r*_1_ = 41 mM^−1^ s^−1^ at 0.5 T MHz and 310 K) than the corresponding hydrogels.

## 1. Introduction

Soft nanostructured materials such as fibers, hydrogels (HGs) and nanogels (NGs) have been identified as promising tools for the development of biomaterials for applications in tissue engineering, targeted drug delivery, biosensors, imaging, gene delivery as well as stimuli-responsive bioactive carriers [1,2,3,4,5,6,7,8,9]. HGs are self-supporting three-dimensional matrices able to entrap high content of water or other biological fluids by non-covalent interactions during the swelling process [10]. Their application in drug delivery is guaranteed by their ability to provide spatial and temporal control in the release of bioactive and therapeutic compounds and by their occurrence in various structures and composition such as injectable forms, thin films, viscous gels, and nanocomposites [2,3,10]. Among these biomedical applications, examples of HGs covalently derivatized or loaded with Gd(III) complexes have been proposed for tissue engineering applications to document the in vivo degradation of implants by magnetic resonance imaging (MRI) [11,12,13]. For instance, Stupp and co-workers described the synthesis of HGs based on peptide amphiphiles (PAs) functionalized with Gd-DOTA-monoamide (DOTA = 1,4,7,10-tetraazacyclododecane-1,4,7,10-tetraacetic acid) complexes and followed their fate over time in tibialis anterior muscle of mouse limbs after implantation [14]. On the other hand, NGs can be defined as nanoparticles with a size in the nano-range compatible with intravenous injection [15]. The major advantages of the nanogel-based systems in comparison to other polymeric nanoparticles characterized by a dense core is that they contain a large amount of water allowing them the capability to encapsulate diverse therapeutic and bioactive molecules, proteins, and metal complexes. One of the approaches used to obtain NGs is by HG submicronization according to top-down methodologies in the presence of stabilizing agents [16]. Following this synthetic protocol, the obtained NGs preserve the hydrated inner network (defined as core) of the HG they are derived from. Another approach describes an ionotropic gelation method that combines a positively charged polysaccharide (chitosan) and an anionic component composed of sodium hyaluronate, tripolyphosphate and anionic Gd(III) complexes to obtain a series of NGs endowed with very high relaxivity values [17]. Mono- and bis-hydrated Gd(III) complexes such as [Gd(DOTA)(H_2_O)]^−^ and [Gd(AAZTA)(H_2_O)_2_]^2−^ (AAZTA = 6-amino-6-methylperhydro-1,4-diazepine-*N*,*N*′,*N*″,*N*″-tetraaceticacid) were incorporated within NGs and relaxivity values up to six times higher than those of the free complexes in aqueous solution were determined [18]. Remarkably, when [Gd(DOTP)]^5−^ (DOTP = 1,4,7,10-Tetraazacyclododecane-1,4,7,10-tetra(methylene phosphonic acid), a Gd(III) complex that lacks metal-bound water molecules (*q* = 0), was confined or used as a cross-linker in these type of NGs, an outstanding relaxivity value of 78.0 mM^−1^ s^−1^ was measured, at 20 MHz and 298 K, nearly 20 times greater than that found for the free complex [19].

**Figure 1:**
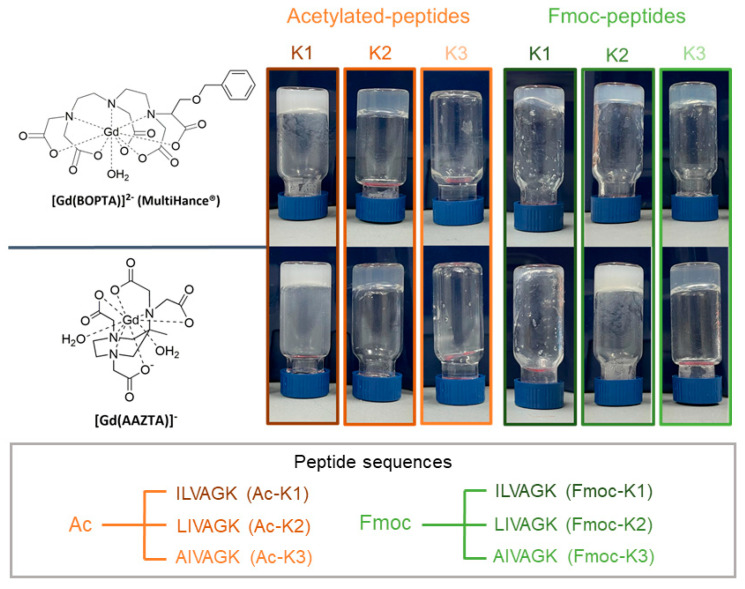
Schematic representation of peptide sequences according to the one code letter practice and of Gd(III) complexes ([Gd(BOPTA)]^2−^) and [Gd(AAZTA)]^−^ used for hydrogel formulations. For each combination, being peptide–Gd(III) chelates, the inverted test tube is also reported.

It is worth noting that among all the proposed matrices, peptides represent a minority and no example of peptide-based NGs [4,20,21,22] has ever been provided for imaging applications; notwithstanding, peptides can be related to several advantages such as high biocompatibility, biodegradability and low cost [23]. Recently, we reported the synthesis, the formulation and the characterization of three peptide-based HGs for tissue engineering applications [24]. The peptide sequences (Figure 1) used to hydrogel fabrication contain five aliphatic residues (including Gly, Ala, Val, Leu and Ile) and a charged one (a Lys residue) at the amidated C-terminus. Moreover, all the peptides are protected at the N-terminus with the Fmoc (fluorenylmethyloxycarbonyl) group, which demonstrated its ability to improve the gelification properties of short and ultrashort peptides. These peptide-based HGs, named as Fmoc-K1, Fmoc-K2 and Fmoc-K3, exhibit a stiffness with storage modulus G’ values between ~500 and ~2500 Pa and very low toxicity (<5–8%) on 3T3 fibroblast and on HaCat cell lines up to 72 h. However, it is important to note that these G′ values are lower than those of some corresponding acetylated (Ac-) peptides (G′ value ~40 kPa), previously studied by Loo et al. as a scaffold for bioprinting applications [25].

In this study, we demonstrated that both Ac- and Fmoc-protected peptide-based hydrogels are able to encapsulate linear ([Gd(BOPTA)]^2−^ and [Gd(DTPA)]^2−^) or mesocyclic ([Gd(AAZTA)]^−^) CAs through ionic and/or hydrophobic interactions. Successively, a nanogel was also obtained by submicronization of the best performing Gd complex-loaded HG. All the supramolecular systems (both HGs and NG) were fully characterized from the structural point of view and their ^1^H NMR relaxometric behavior was investigated as a function of the applied magnetic field strength.

## 2. Results and Discussion

### 2.1. Formulation and Characterization of HGs Encapsulating Gd Complexes

Empty HGs based on acetyl (Ac-) or fluorenylmethyloxycarbonyl (Fmoc-) K1, K2 and K3 peptides were recently investigated as three-dimensional matrices for cell adhesion and proliferation [24]. Due to the presence of a charged lysine residue in their sequence, these peptides are here selected as starting models for improving the electrostatic interactions and promoting the physical encapsulation of *T*_1_-CAs such as [Gd(AAZTA)]^−^ [26,27] and [Gd(BOPTA)]^2−^ [28], which contain one and two negative charges, respectively. The choice of these two Gd complexes comes from the purpose of obtaining both the best encapsulation ratio and the highest relaxivity (*r*_1_) value. This latter is a parameter that defines the efficacy of any paramagnetic complex agent to induce a change in the water protons relaxation rate (*R*_1_) per unit concentration (mmol L^−1^) of metal ion [29,30]. Regarding the first point, two contributes were considered: (i) the electrostatic interactions between the peptide and the two differently charged complexes and (ii) the possibility to introduce further interactions lead by the aromatic moieties of the components. Indeed, the benzene ring on the backbone of BOPTA ligand could establish π–π stacking interactions with the Fmoc group on the peptide derivatives. To study the influence of these interactions on the relaxivity, Fmoc-K2 hydrogels loaded with [Gd(DTPA)]^2−^ were also prepared and studied. On the other hand, [Gd(AAZTA)]^−^ was chosen because of the high relaxivity value (*r*_1_=6.6 mM^−1^ s^−1^ at 32 MHz and 298 K) due to the presence of two inner sphere water molecules coordinated to the metal ion. Recent studies evidenced that the formulation of empty HGs of these cationic peptides is easily obtained by adding 50 µL of 0.1 mol L^−1^ phosphate buffer to an aqueous suspension (300 µL of the peptide solution) of each peptide at a concentration of 2 wt% [24]. In this procedure, the hydrogel formation is triggered by the phosphate buffer, which has probably a role in decreasing the electrostatic repulsions between the positive charges of lysine residues. The rheological characterization of these two classes of peptides evidenced a different mechanical rigidity, with a higher stiffness for some of the Ac-derivatives with respect to Fmoc-ones. It is easy to infer that the stiffness of each formulation can significantly affect the application of the final biomaterial.

For [Gd(BOPTA)]^2−^- and [Gd(DTPA]^2−^-loaded HGs, due to the presence of residual negative charges on the metal complex, we observed the spontaneous formation of the gel by simply adding 50 µL of the 30 mmol L^−1^ complex solution to the 2 wt% peptide one. On the other hand, the [Gd(AAZTA)]^−^-loaded HGs formulations (2 wt%) were obtained by dissolving the peptide powder in a 5 mmol L^−1^ complex solution, followed by the addition of 50 µL of phosphate buffer. This last step was necessary since the single residual negative charge of [Gd(AAZTA)]^−^ was not enough to prompt the gelation process.

The capability of each peptide to gel also in the presence of the Gd(III) complex was evaluated by the inverted test tube (Figure 1 and Figure S4C). As observed in the pictures, not all the peptides keep their propensity to gel: Ac-K3 does not form gels with both complexes and Fmoc-K1 is not able to gel in the presence of [Gd(AAZTA)]^−^ and seems to form very soft, not completely supporting gels with [Gd(BOPTA)]^2−^. This latter evidence is not surprising since Fmoc-K1 was previously identified as the softer hydrogel of the series with a storage module (G′) of 557 Pa [24]. However, the encapsulation of [Gd(BOPTA)]^2−^ in Fmoc-K1 is not completely forbidden thanks to the interactions between the aromatic ring of the chelating agent and the Fmoc group. Instead, the inability of Ac-K3 to gel could be probably explained based on its different peptide sequence, in which the presence of an alanine residue in place of a leucine or an isoleucine, together with the absence of the fluorenyl group, hinders a good packaging. The stability of filled HGs was also evaluated over time (up to 30 days) by incubating them in the Ringer’s solution at 37 °C and by evaluating their weight loss. Results, reported in Table 1 as ΔW(%), indicate a low weight loss for all the hydrogels (<7.79%), thus pointing to a high stability.

### 2.2. Structural Characterization of Hydrogels: FTIR, SEM and Rheology

**Figure 2:**
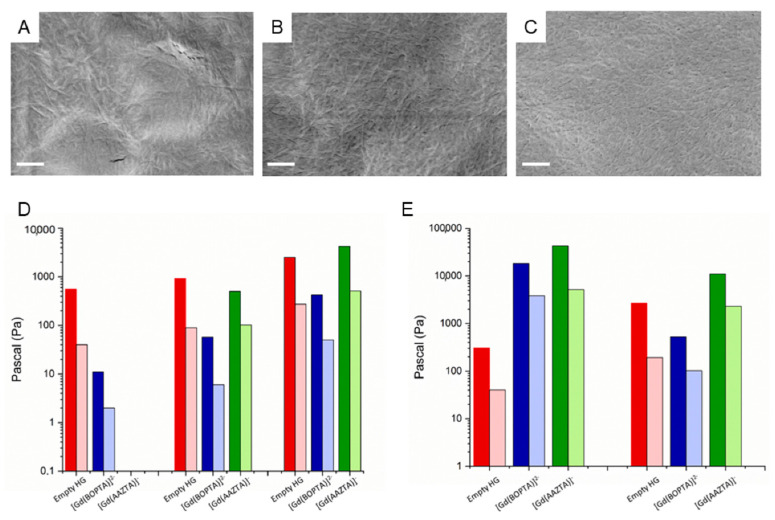
Structural characterization of hydrogels filled with Gd complexes. SEM micro-photos of Fmoc-K1 (**A**), Fmoc-K2 (**B**), and Fmoc-K3 (**C**) loaded with [Gd(BOPTA)]^2−^ (scale bar is 500 nm). Rheological histogram analysis performed on hydrogels loaded with Gd complexes. (**D**) Fmoc-peptides; (**E**) Ac-peptides. Graph report both G′ (dark bar) and G″ (light bar) moduli of each time sweep experiment (20 min, strain of 0.1%, frequency 1 Hz). Values are expressed as a Pascal (Pa) logarithmic scale. Time sweep measurements are not in triplicate.

Further structural characterization of paramagnetic hydrogels was assessed by Fourier transmission infrared (FTIR) and scanning electron microscopy (SEM) techniques. Selected FTIR spectra of Gd complexes loaded into hydrogels and of the corresponding empty ones are reported in Figure S5. From the inspection of the spectra, a common transmittance profile can be observed for all the examined samples. In detail, IR profiles are dominated by two main signals, typically observed in the case of aggregates rich in β-sheet structures. The first very broad signal is detectable in the amide A region (~3500–3300 cm^−1^) and the second one in the amide I region (1700 to 1600 cm^−1^). More precisely, the band around 3395 cm^−1^ is originated by the NH stretching vibrations polarized along the N-H bond and by the O-H stretching occurring between the hydrogel matrix and the surrounding water. The high intensity of this band is indicative of the extended network of inter- and intramolecular hydrogen bonds existing in the supramolecular structure. On the other hand, the band around 1650 cm^−1^ is due to the C=O stretching vibration and used to confirm the presence of β-sheet structures. Micrographs of selected samples (Fmoc-K1, Fmoc-K2 and Fmoc-K3 encapsulating [Gd(BOPTA)]^2−^) are reported in Figure 2A–C. SEM images of the xerogels show fibrillary interconnected networks, typically observed for peptide-based hydrogels. By comparison with the corresponding empty hydrogels, it can be concluded that the insertion of the metal complex does not affect the overall morphology. Similar results were obtained also for Fmoc-peptide-based hydrogels encapsulating [Gd(AAZTA)]^−^ and [Gd(DTPA]^2−^ (see Figure S6) and for Ac-peptide HGs encapsulating the same complexes. A rheological analysis was carried out to assess the effect of Gd(III) complexes physical entrapment on the mechanical properties of the final matrix. A rotational controlled stress rheometer was used, and the experiments were carried out in the linear viscoelastic region after a preliminary parameter evaluation.

Rheological results, collected in Figure 2D,E and Figure S4D, are reported in terms of G′ (storage modulus) and G″ (loss modulus). All the acquired time sweep oscillatory profiles (1.0 Hz and 0.1% strain, 20 min) are in Figures S7 and S8 and viscoelastic parameters (G′, G″ and tangent of the phase angle, tanδ) are grouped in Table 1. All the samples keep the gel state, possessing values of tanδ > 1 and G′ > G″, consistent with materials that behave similarly to an elastic solid. The [Gd(BOPTA)]^2−^ entrapment in Fmoc-K series generates a sensible decrease of the G′ modulus, particularly relevant for Fmoc-K1 (around 50-fold decrease), which is the softer peptide of the series. This trend is also visible in the tanδ decrease for each couple of empty and [Gd(BOPTA)]^2−^ filled Fmoc-K-based hydrogels. This general behaviour could be probably explained considering the chemical structure of the Gd(III) chelate. The presence of an aromatic moiety in the complex and its overall steric hindrance is probably able to alter the aggregative interaction pathway of Fmoc-hexapeptides, reducing the Fmoc–Fmoc interactions responsible for the self-assembling phenomena. This explanation is also supported by the inverse trend observed in Ac-K peptides, for which the G’ values increase with the encapsulation of [Gd(BOPTA)]^2−^ (increase of around 60-fold for Ac-K1).

The [Gd(AAZTA)]^−^ entrapment within the hydrogel matrices induces an increase of the mechanical rigidity in Fmoc-K3 (G′ value from 2526 Pa to 4210 Pa for empty and filled, respectively), a decrease of G′ (~2-fold) in Fmoc-K2 (500 Pa respect to 925 Pa previously measured for the empty HG) and it prevents the formation of hydrogels in Fmoc-K1. As observed for [Gd(BOPTA)]^2−^, [Gd(AAZTA)]^−^ also causes an increase of the G′ values in the Ac-K series, which is especially evident for Ac-K1, with an increase of G′ of 140-fold. Likely, this exceptional increase can be explained by taking into account the three-dimensional distribution of the complexes in the supramolecular space.

### 2.3. Hydrogel Relaxivity Studies

**Figure 3:**
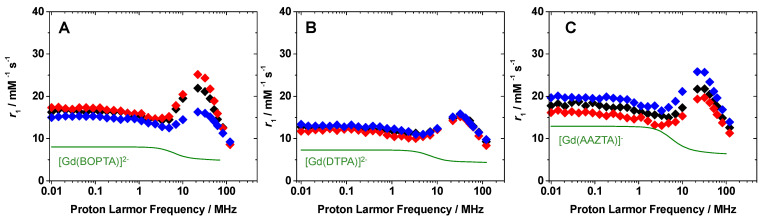
^1^H NMRD profiles of Fmoc-K2+[Gd(BOPTA)]^2−^ (**A**), Fmoc-K2+[Gd(DTPA)]^2−^ (**B**) and Fmoc-K2+[Gd(AAZTA)]^−^ (**C**) at 283 (blue), 298 (black) and 310 K (red). The profiles of the corresponding chelates in aqueous solution are also reported as solid lines.

**Figure 4:**
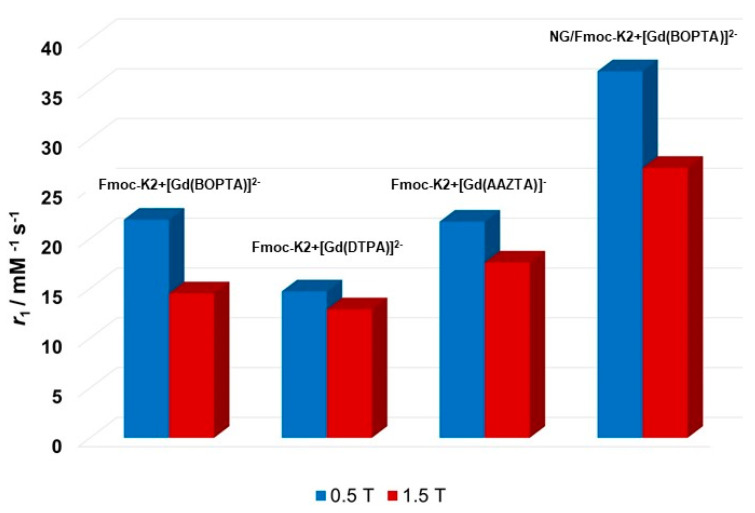
Comparison of the *r*_1_ values calculated at 0.5 and 1.5 T (298 K) for Fmoc-K2+[Gd(BOPTA)]^2−^, Fmoc-K2+[Gd(DTPA)]^2−^, Fmoc-K2+[Gd(AAZTA)]^−^ and NG/Fmoc-K2+[Gd(BOPTA)]^2−^. Relaxometric measurements are not in triplicate.

The relaxometric properties of Fmoc-K2 hydrogels were further investigated by measuring the relaxivity values as a function of the applied magnetic field strength in the 0.01–120 MHz range, at three different temperatures (283, 298 and 310 K) and these were compared with those of the free chelates in aqueous solution at 298 K and neutral pH. In principle, the values of *r*_1_ receive contributions from three different mechanisms: the inner sphere mechanism (*r*_1_^IS^) generated by the direct dipolar interaction of the coordinated water molecules with the metal ion; the second sphere mechanism (*r*_1_^SS^), promoted by the interaction of the water molecules hydrogen-bonded to the polar groups of the ligand with the paramagnetic site; the outer sphere contribution (*r*_1_^OS^) defined by the weak interactions of rapidly diffusing bulk water molecules next to the complex. Typically, the first mechanism dominates the observed relaxivity and mainly depends on the hydration state of the chelate (*q*), as well as the residence lifetime (*τ*_M_) of the metal-coordinated water molecule(s), the rotational correlation time of the probe (*τ*_R_) and the electronic relaxation times [31]. The ^1^H NMRD profile of Fmoc-K2+[Gd(BOPTA)]^2−^ showed a field-dependence relaxivity typical of probes containing Gd(III) chelates with restricted mobility, with a marked hump centered around 20 MHz [18]. The strong π–π interactions of the aromatic group of the complex with the Fmoc functionality of the peptide-based hydrogel are responsible of both the shape of the profile and the relatively high *r*_1_ values observed. At 20 MHz and 298 K, the relaxivity of the hydrogel is ca. five times higher than that of mono-hydrated [Gd(BOPTA)]^2−^ free complex (Figure 3A and Figure 4). Surprisingly, the relaxivity of the hydrogel increases with the temperature, a behavior opposite to that typically observed of negatively charged Gd(III) chelates [32]. This suggests the occurrence of a slow water exchange process, probably due to a reduced diffusion of water inside the gelled matrix (Figure 3A). In order to confirm the importance of the interactions between aromatic moieties in Fmoc-K2+[Gd(BOPTA)]^2−^ hydrogels, the corresponding sample containing [Gd(DTPA)]^2−^ was also analyzed. The Fmoc-K2+[Gd(DPTA)]^2−^ hydrogel shows a different ^1^H NMRD profile with a less pronounced peak at high magnetic fields and lower relaxivity values (Figure 3B and Figure 4), likely due to a weaker interaction of the complex with the peptide matrix. Nevertheless, the relaxivity at 20 MHz and 298 K is ca. three times higher than that of free [Gd(DTPA)]^2−^ in aqueous solution. Furthermore, although *r*_1_ does not increase with increasing temperature, as observed for Fmoc-K2+[Gd(BOPTA)]^2−^, the water exchange regime is still slow and limited by the diffusion processes.

Finally, ^1^H NMRD profiles of the same peptide hydrogel entrapping the bis-hydrated [Gd(AAZTA)]^−^ complex were measured. The increment of the *r*_1_ values at 20 MHz with respect to the pure complex is comparable to that observed for Fmoc-K2+[Gd(BOPTA)]^2−^, because of the presence of two inner sphere water molecules coordinated to Gd^3+^. Moreover, as *r*_1_ decreases with increasing temperature, we can conclude that the water exchange process is fast enough not to limit relaxivity (Figure 3C).

**Figure 5:**
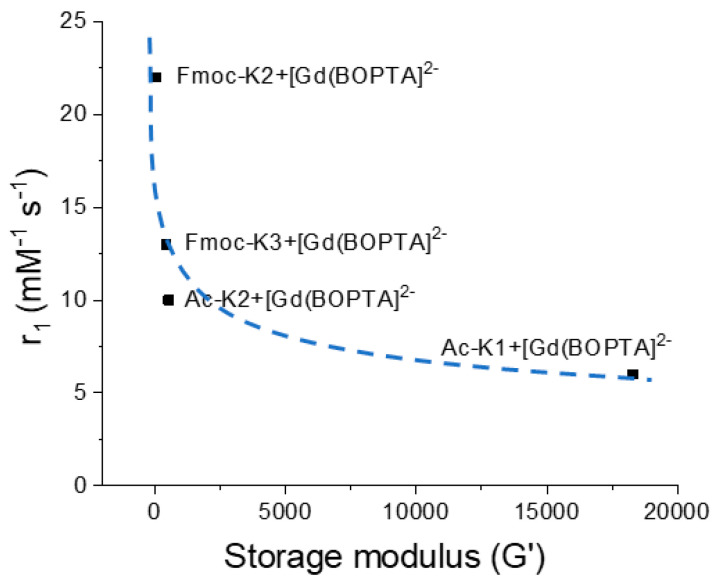
Dependence of *r*_1_ values (20 MHz and 298 K) on the storage modulus (G′) of hydrogels based on [Gd(BOPTA)]^2−^.

Some hypotheses could explain the different water dynamics in the various hydrogels: (i) localization of the complexes to sites of the hydrogel characterized by different water accessibilities; (ii) different organization of the water network in the three hydrogels; (iii) alteration of the rigidity of the matrix in the presence of different chelates, as indicated by the G′ values shown in Table 1. One or more of these conditions can occur simultaneously and result from the high complexity of these systems. Under these conditions, it has no physical meaning to try to apply well-known models, which describe the paramagnetic relaxation and allow assessing the molecular parameters responsible for the relaxivity values. Moreover, the modification of the peptide structure, responsible for the gelation, can dramatically change the relaxometric properties of the final hydrogel. In fact, a marked decrease of *r*_1_ takes place moving from Fmoc-K2 to the more rigid Fmoc-K3 hydrogels (Figure 5 and Figure S9). A similar behavior was observed also for the peptide functionalized with acetyl groups (Ac-K1 and Ac-K2), where only a weak interaction occurs between the [Gd(BOPTA)]^2−^ complexes and the polymeric matrix (Figure 5 and Figure S10). Indeed, in Ac-K1 and Ac-K2 peptidic variants the aromatic fluorenyl group is replaced by the acetyl group, which cannot provide π–π stacking interactions with the aromatic ring of the BOPTA chelating agent. The comparison between the encapsulation of DTPA and BOPTA Gd complexes into the hydrogel has clearly highlighted the relevance of such interactions in enhancing the *r*_1_ value. The decrease of *r*_1_ with an increasing storage modulus of HGs encapsulating [Gd(BOPTA)]^2−^ is clearly highlighted in Figure 5.

### 2.4. Nanogel Formulation and Structural and Relaxivity Characterization

**Figure 6:**
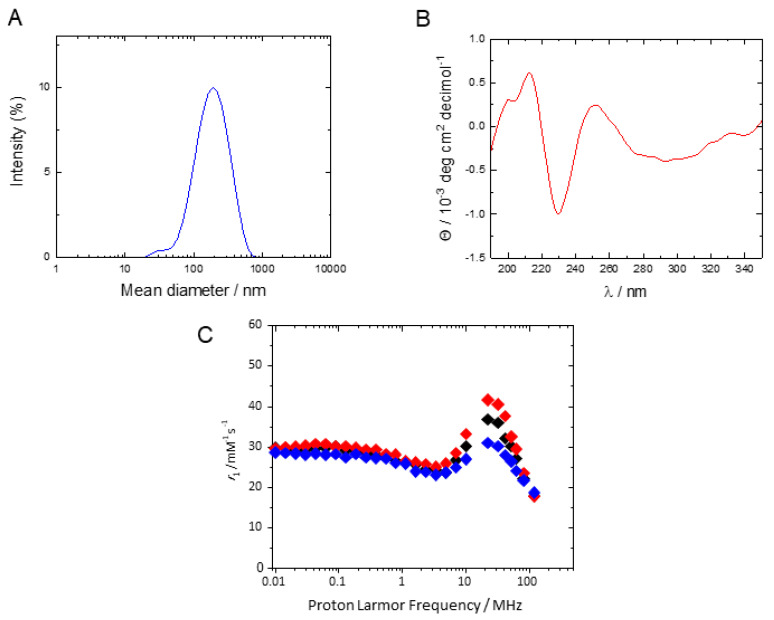
Structural and relaxometric characterization of Fmoc-K2 NG loaded with [Gd(BOPTA)]^2−^. (**A**) CD profile of the NG solution recorded in the range between 350 and 190 nm; (**B**) Dynamic light scattering profile and the paramagnetic NG; (**C**) ^1^H NMRD profile of the nanogel at 283 (blue), 298 (black) and 310 K (red).

In view of the interesting relaxometric properties of the Fmoc-K2+[Gd(BOPTA)]^2−^ hydrogel, this sample was treated with a submicronization process and converted into the corresponding nanogel (NG/Fmoc-K2+[Gd(BOPTA)]^2−^) using the top-down approach. Submicronization of the hydrogel was carried out in presence of two commercial surfactants TWEEN^®^ 85 (Polyoxyethylenesorbitan Trioleate) and SPAN^®^ 85 (Sorbitane trioleate), which have the role to stabilize the supramolecular formulation. The 89/11 (*w*/*w*) ratio between TWEEN^®^85 and SPAN^®^85 was chosen to obtain an HLB value of 10, which previously emerged as the optimal one for similar peptide-based NG formulations [22]. At the end of the preparation the resulting NGs were purified from free Gd complex by gel filtration and the encapsulated fraction of the complex was estimated by inductively coupled plasma-mass spectrometry (ICP-MS). The size and the stability of the NG formulation were assessed over time using the dynamic light scattering (DLS) technique, by measuring the mean diameter up to seven days. he mean diameter and the polydispersity index reported in Figure 6A (~190 nm and 0.272, respectively) are compatible with the possibility of a systemic administration. In order to obtain structural information on the resulting nanogel, circular dichroism (CD) studies were performed. The shape of the CD profile upholds that the structuration of the HG is preserved also in its nano-derivative (Figure 6B) [24]. The positive band, with a maximum around 212 nm, is attributable to n→π* transitions occurring in the β-sheet organization, while the one around 250 nm is associated with the π–π* transition of fluorenyl moiety. Moreover, the nanogel presents a unique negative band at ~230 nm, which has been demonstrated to be relative to the presence of strongly twisted β-sheets in which both the inter-β-sheet distance and the solvent exposure increase [33]. This higher accessibility to water is well justified by the concept of nanogels itself, characterized by the retention of the fibrillary network of HGs and by the increase of the surface area.

The relaxometric properties of an aqueous suspension of nanogel containing 0.4 mmol L^−1^ of [Gd(BOPTA)]^2−^ were investigated. The shape of the ^1^H NMRD profile and the temperature dependence of the *r*_1_ values are comparable to those observed for the corresponding hydrogel (Figure 6C). A strong limitation of the mobility of the complex in interaction with the aromatic functionalities of the peptide matrix is still evident after the submicronization process of the hydrogel and the water exchange process follows a slow regime. Furthermore, it is important to note that the maximum relaxivity at 20 MHz and 298 K is 36.8 mM^−1^ s^−1^, a value higher than that measured for the corresponding hydrogel, probably because of a different degree of rotational freedom of the complexes within the polymeric matrix.

## 3. Materials and Methods

Protected N^α^-Fmoc-amino acid derivatives, coupling reagents and Rink amide MBHA (4-methylbenzhydrylamine) resin are commercially available from Calbiochem-Novabiochem (Laufelfingen, Switzerland). All other chemicals are commercially available from Merck (Milan, Italy), Fluka (Bucks, Switzerland), or LabScan (Stillorgan, Dublin, Ireland) and unless stated otherwise, they were used as delivered. [Gd(BOPTA)]^2−^ (Gadobenate dimeglumine, MultiHance) was kindly provided by Bracco Imaging S.p.A. [Gd(AAZTA)]^−^ was synthesized as reported previously [26]. Peptide solutions were prepared by weight using double distilled water.

To promote the complexation in [Gd(DTPA)]^2−^, Diethylenetriaminepentaacetic acid (DTPA) (20 mg, 0.05 mmol) was dissolved in 2 mL of pure water in the presence of a molar excess of GdCl_3_·6H_2_O. The pH of the solution was corrected to 6 with NaOH 0.1 M and the reaction was maintained under stirring at room temperature for 1h. Then, the pH was increased to 9.5 to promote the precipitation of unreacted Gd^3+^ that was filtered out, and the pH of the solution was corrected to 7 with diluted HCl (0.1 M).

### 3.1. Peptide Solid Phase Synthesis

All the peptide derivatives were synthesized using standard solid-phase peptide synthesis (SPPS) protocols according to Fmoc/tBu strategy as previously described [24]. Briefly, the solid support (Rink amide MBHA resin, substitution grade 0.73 mmol/g) was swelled in dimethylformamide (DMF) under stirring for 30 min. Successively, the Fmoc protecting group was removed from the resin using a piperidine/DMF mixture (30/70, *v*/*v*). After the deprotection step, amino acids were sequentially coupled on the support by adding to the reactor a 2-fold molar excess of Fmoc-protected amino acid, 1-hydroxybenzotriazole (HOBt) and benzotriazol-1-yl-oxytris-pyrrolidino-phosphonium (PyBOP), as well as a 4-fold molar excess of diisopropylethylamine (DIPEA) in DMF/NMP 1/1 (*v*/*v*). Each coupling was repeated twice for 40 min under stirring at room temperature. The Fmoc-derived peptides (Fmoc-ILVAGK (Fmoc-K1), Fmoc-LIVAGK (Fmoc-K2) and Fmoc-AIVAGK (Fmoc-K3)) were directly cleaved from the resin using a TFA (trifluoroacetic acid)/TIS (triisopropylsilane)/H_2_O (92.5/5/2.5 *v*/*v*/*v*) mixture for 2 h. Instead, Ac-K1, Ac-K2 and Ac-K3 peptides were obtained by removing the Fmoc group from the last amino acid and acetylating twice the N-terminus with a solution of pyridine/acetic anhydride (4/4.7 *v*/*v*) in DMF (each treatment for 10 min). After the acetylation step, these peptides were cleaved from the resin according to the above-described procedure. All the peptides were then precipitated with cold ether and freeze-dried for three times.

Purification was achieved using preparative RP-HPLC with a LC8 Shimadzu HPLC system (Shimadzu Corporation, Kyoto, Japan) equipped with a UV lambda-Max Model 481 detector, using a Phenomenex (Torrance, CA, USA) C_18_ column. H_2_O/0.1% TFA (A) and CH_3_CN/0.1% TFA (B) were used as elution solvents with the concentration of (B) increasing from 30 to 80% over 30 min at a flow rate of 20 mL/min. The purity of Ac-K1, Ac-K2 and Ac-K3 products was confirmed by analytical RP-HPLC-MS analysis (Figures S1–S3) performed by using a Finnigan Surveyor MSQ single quadrupole electrospray ionization detector (Finnigan/Thermo Electron Corporation, San Jose, CA, USA), with a C_18_-Phenomenex column eluting with (A) and (B) at a flow rate of 1 mL/min. The analytical method is characterized by a gradient from 5 to 70% of (B) occurring over 10 min. The identity of peptides was assessed by MS spectrometry conducted by the use of a LTQ XL Linear Ion Trap Mass Spectrometer, ESI source (Figures S1–S3).

Ac-K1: t_R_ = 11.62 min, MS (ESI+): m/z 640.82 calcd. for C_30_H_56_N_8_O_7_: [M+H^+^] = 641.4 u.m.a.

Ac-K2 t_R_ = 11.51 min, MS (ESI+): m/z 640.82 calcd. for C_30_H_56_N_8_O_7_: [M+H^+^] = 641.5 u.m.a.

Ac-K3: t_R_ = 10.13 min, MS (ESI+): m/z 598.74 calcd. for C_27_H_50_N_8_O_7_: [M+H^+^] = 599.4 u.m.a.

### 3.2. Preparation of Peptide Hydrogels Loaded with Gd-Complexes

Peptide-based HGs loaded with different Gd complexes were prepared at the final Gd(III) concentration of 4.28 mmol L^−1^. To this effect, for [Gd(BOPTA)]^2−^ and [Gd(DTPA)]^2−^-loaded HGs, peptide solutions were prepared at a concentration of 2 wt% by dissolving 6 mg of each lyophilized peptide into 300 µL of bidistilled water; then, 50 µL of an aqueous solution of the Gd complex (at a concentration of 30 mmol L^−1^) was added to the suspension. The resulting solution was sonicated for 5 min and left to gelatinate at room temperature. Since [Gd(AAZTA)]^−^ presents only one negative charge on its structure, one less than the other two linear complexes previously mentioned, [Gd(AAZTA)]^−^-loaded HGs were formulated by a different procedure. In more detail, 6 mg of the lyophilized peptide powder were first dissolved into 300 µL of an aqueous solution of [Gd(AAZTA)]^−^ at a concentration of 5.0 mmol L^−1^, and then, to better balance electrostatic forces of the HG inner structure, 50 µL of 0.1 mol L^−1^ phosphate-buffered solution were added. This procedure allowed the gel formation.

### 3.3. Hydrogel Stability Studies

The determination of the HG degradation profile was performed by an in vitro stability assay, evaluating the percentage weight loss of the matrices. Freshly formed HGs (300 μL) were weighted (W_o_) and then incubated at 37 °C with 1.0 mL of Ringer’s solution (8.6 mg of NaCl, 0.30 mg of KCl and 0.33 mg of CaCl_2_) [34]. After 20 days, the Ringer’s solution was removed and HGs were weighted again (W_t_). The degradation degree was expressed as the percentage ratio (∆W) between the HG weight before (W_o_) and after (W_t_) the treatment using the following formula (1):

(1) $$\Delta W=\left(1-\frac{\mathrm{Wt}}{\mathrm{Wo}}\right)*100$$

### 3.4. Scanning Electron Microscopy (SEM)

Morphological analysis of derivative xerogels was carried out by a field emission scanning electron microscope (PhenomXL, Alfatest). A total of 10 μL of peptide HG was drop-casted on an aluminum stub and air-dried. A thin coat of gold and palladium was sputtered at a current of 25 mA for 75 s. The sputter-coated samples were then introduced into the specimen chamber and the images were acquired at an accelerating voltage of 10 kV, spot 3, through the Secondary Electron Detector (SED).

### 3.5. Fourier Transform Infrared Spectroscopy (FT-IR)

Fourier transform infrared spectra of Gd complex-loaded hydrogels prepared as described above were performed on a Jasco FT/IR 4100 spectrometer (Easton, MD) in an attenuated total reflection (ATR) mode and using a Ge single crystal at a resolution of 4 cm-1as previously reported [24].

### 3.6. Rheological Studies

Rheological properties of HGs loaded with each Gd-complex were evaluated using a rotational controlled stress rheometer (Malvern Kinexus) using a 15 mm flat-plate geometry (PU20:PL61). Freshly prepared hydrogel samples (400 μL) at the concentration of 2.0 wt% were tested. Each experiment was performed at 25 °C using a humidity chamber and a gap of 1 mm. Preliminary dynamic rheological tests were carried out in order to identify the regime of linear viscoelasticity. The viscous elastic region was determined by the oscillatory frequency (0.1–100 Hz) and the strain sweep (0.01–100%). A time-sweep oscillatory evaluation test (using a constant 0.1% strain and 1 Hz frequency) was then performed for 20 min. Results are reported in Pascal (Pa) as shear storage or elastic modulus (G′) and shear loss or viscous modulus (G″).

### 3.7. Nanogel Formulation

[Gd(BOPTA)]^2−^-loaded nanogel was obtained as previously described according to the top-down method [22]. Briefly, 350 µL of [Gd(BOPTA)]^2−^-loaded Fmoc-K2 gel disk (1.7% wt), prepared into a silicone mold, was added to 1.650 mL of a water suspension of two surfactants, TWEEN^®^ 85 (Polyoxyethylenesorbitan Trioleate) and SPAN^®^ 85 (Sorbitane trioleate) at a *w*/*w* ratio of 89/11 (2.34·10^−5^ total mol of surfactants). The two surfactants were thus combined to form an HLB (hydrophilic lipophilic balance) value of 10. Successively, the sample was first homogenized at 35,000 min^−1^ for 5 min, and then subjected to tip-sonication for 5 min at 9 W. Purification of the NG from free Gd complexes was achieved by gel filtration on a pre-packed Sephadex G-50 column and the amount of encapsulated complex was quantified by inductively coupled plasma-mass spectrometry (ICP-MS).

### 3.8. Dynamic Light Scattering (DLS) Measurements

Mean diameters and diffusion coefficients (*D*) of CA-filled NGs were estimated by DLS using a Zetasizer Nano ZS (Malvern Instruments, Westborough, MA, USA). Analogously, nanogel stability over time was checked measuring the mean diameter into different time points up to seven days. Instrumental settings for the measurements are a backscatter detector at 173° in automatic modality mode, at room temperature and using a disposable sizing cuvette as a cell. DLS measurements in triplicate were carried out on aqueous samples after centrifugation at room temperature at 13,000 rpm for 5 min.

### 3.9. Circular Dichroism (CD) Studies

Far-UV CD spectra of [Gd(BOPTA)]^2−^ loaded Fmoc-K2 nanogel were collected with a Jasco J-810 spectropolarimeter equipped with a NesLab RTE111 thermal controller unit using a 0.5 mm quartz cell at 25 °C. Other experimental settings were the following: scan speed = 50 nm/min, sensitivity = 50 mdeg, time constant = 16 s, bandwidth = 1 nm, response = 2 s and data pitch = 1 nm. The spectrum was recorded in triplicate from 350 to 190 nm, subtracted from the blank and adjusted for the concentration.

### 3.10. Relaxometric Characterization

1/*T*_1_ ^1^H nuclear magnetic relaxation dispersion (NMRD) profiles were collected with a fast-field cycling (FFC) Stelar SmarTracer Relaxometer over a continuum of magnetic field strengths from 0.00024 to 0.25 T. The relaxometer operates under computer control with an absolute uncertainty in 1/*T*_1_ of ±1%. Additional data in the 0.5–3.0 T were obtained with a High Field Relaxometer (Stelar) equipped with the HTS-110 3T Metrology cryogen-free superconducting magnet. The data were obtained by using the standard inversion recovery sequence (20 experiments, 2 scans) with a 90° pulse width of 3.5 μs, and the reproducibility of the data was within ±0.5%. The NMRD profiles were collected at 283, 298 and 310 K.

## 4. Conclusions

Hydrogels based on cationic peptide sequences (derivatized at their N-terminus with the acetyl or the fluorenyl group) demonstrated the capability of stably encapsulate linear or mesocyclic Gd complexes such as [Gd(BOPTA)]^2−^, [Gd(DTPA)]^2−^, [Gd(AAZTA)]^−^, having one or two residual negative charges. When formed, the physical encapsulation of the complex does not significantly alter the hydrogel morphology, in which the fibrillary network is kept. On the other hand, rheological characterization highlighted how the encapsulation of a cargo within the gel can allow an increase or a decrease of the matrix rigidity as a consequence of the non-covalent interactions (π–π stacking, hydrogen bonding and electrostatic interactions). As expected, the CAs loaded into the macroscopic hydrogel exhibit a relaxivity value higher than the corresponding free Gd complex (up to five-fold). Beyond the typical relaxometric parameters (τ_R_, τ_M_ and *q*), which affect the relaxivity, the *r*_1_ value seems to be influenced by other key factors such as the mechanical properties of the hydrogel, the interactions between the complex and the peptide matrix and the water accessibility to the complex within the hydrogel. As a whole, the cited parameters must be deeply investigated and optimized to improve the relaxivity performance of the resulting CA. The relaxometric study performed on the injectable nanoparticles, obtained from the hydrogel submicronization, evidenced a reduced mobility of the complex into the peptide matrix and a slow regime of the water exchange process. The higher relaxivity at 20 MHz and 298 K (*r*_1_ = 36.8 mM^−1^ s^−1^) measured for the nanogel with respect to the corresponding hydrogel can be probably attributed to the different degree of rotational freedom of the complexes within the polymeric matrix.

## Figures and Tables

**Table 1:** Degradation degree, expressed as percentage ratio (∆W), storage modulus (G′), loss modulus (G″) and Tanδ for the different paramagnetic formulations. Rheological parameters of empty hydrogels are also reported for comparison.

	Sample	ΔW (%)	G′ (Pa)	G″ (Pa)	Tan (δ)
**Fmoc-K1**	Fmoc-K1	---	557	40	13.9
	Fmoc-K1 + [Gd(BOPTA)]^2−^	3.74	11	2	5.5
	Fmoc-K1 + [Gd(AAZTA)]^−^		-	-	-
**Fmoc-K2**	Fmoc-K2	---	925	89	10.4
	Fmoc-K2 + [Gd(BOPTA)]^2−^	2.65	57	6	9.5
	Fmoc-K2 + [Gd(AAZTA)]^−^	4.91	500	102	4.9
	Fmoc-K2 + [Gd(DTPA)]^2−^	---	36	3	12
**Fmoc-K3**	Fmoc-K3	---	2526	273	9.2
	Fmoc-K3 + [Gd(BOPTA)]^2−^	1.08	425	51	8.3
	Fmoc-K3 + [Gd(AAZTA)]^−^	0.01	4210	508	8.3
**Ac-K1**	Ac-K1	---	306	40	7.6
	Ac-K1 + [Gd(BOPTA)]^2−^	0.20	18,280	3826	4.8
	Ac-K1 + [Gd(AAZTA)]^−^	0.67	42,583	5182	8.2
**Ac-K2**	Ac-K2	---	2677	192	13.9
	Ac-K2 + [Gd(BOPTA)]^2−^	7.79	520	102	5.1
	Ac-K2 + [Gd(AAZTA)]^−^	2.81	11,018	2304	4.8

## Data Availability

Data is contained within the article and Supplementary Materials.

## Supplementary Materials

The following supporting information can be downloaded at: https://www.mdpi.com/article/10.3390/ph15121572/s1, Figures S1–S3: Chemical structures, RP-HPLC chromatograms and ESI mass spectra of peptides. Figure S4: Characterization of [Gd(DTPA)]^2−^ loaded Fmoc-K2 hydrogel. Figure S5: FTIR spectra of hydrogels. Figure S6: SEM micro-photos of Fmoc-K2 and Fmoc-K3 (B) loaded with [Gd(AAZTA)]^−^. Figures S7 and S8: Rheological profiles of time sweep measurements. Figures S9 and S10: ^1^H NMRD profiles of Fmoc-K3, Ac-K1 and Ac-K2 hydrogels loaded with Gd-complexes.

## References

[1] Roth-Konforti M.E., Comune M., Halperin-Sternfeld M., Grigoriants I., Shabat D., Adler-Abramovich L. (2018). UV Light-Responsive Peptide-Based Supramolecular Hydrogel for Controlled Drug Delivery. Macromol. Rapid Commun..

[2] Dreiss C.A. (2020). Hydrogel design strategies for drug delivery. Curr. Opin. Colloid Interface Sci..

[3] Dimatteo R., Darling N.J., Segura T. (2018). In situ forming injectable hydrogels for drug delivery and wound repair. Adv. Drug Deliv. Rev..

[4] Gallo E., Diaferia C., Rosa E., Smaldone G., Morelli G., Accardo A. (2021). Peptide-Based Hydrogels and Nanogels for Delivery of Doxorubicin. Int. J. Nanomed..

[5] Karavasili C., Panteris E., Vizirianakis I.S., Koutsopoulos S., Fatouros D.G. (2018). Chemotherapeutic Delivery from a Self-Assembling Peptide Nanofiber Hydrogel for the Management of Glioblastoma. Pharm. Res..

[6] Huettner N., Dargaville T.R., Forget A. (2018). Discovering Cell-Adhesion Peptides in Tissue Engineering: Beyond RGD. Trends Biotechnol..

[7] Saunders L., Ma P.X. (2019). Self-Healing Supramolecular Hydrogels for Tissue Engineering Applications. Macromol. Biosci..

[8] Aviv M., Halperin-Sternfeld M., Grigoriants I., Buzhansky L., Mironi-Harpaz I., Seliktar D., Einav S., Nevo Z., Adler-Abramovich L. (2018). Improving the Mechanical Rigidity of Hyaluronic Acid by Integration of a Supramolecular Peptide Matrix. ACS Appl. Mater. Interfaces.

[9] Vieira V.M.P., Lima A.C., De Jong M., Smith D.K. (2018). Commercially Relevant Orthogonal Multi-Component Supramolecular Hydrogels for Programmed Cell Growth. Chem. Eur. J..

[10] Chamkouri H., Chamkouri M. (2021). A Review of Hydrogels, Their Properties and Applications in Medicine. Am. J. Biomed. Sci. Res..

[11] Berdichevski A., Yameen H.S., Dafni H., Neeman M., Seliktara D. (2015). Using bimodal MRI/fluorescence imaging to identify host angiogenic response to implants. Proc. Natl. Acad. Sci. USA.

[12] Fragai M., Ravera E., Tedoldi F., Luchinat C., Parigi G. (2019). Relaxivity of Gd-Based MRI Contrast Agents in Crosslinked Hyaluronic Acid as a Model for Tissues. ChemPhysChem.

[13] Diaferia C., Gianolio E., Accardo A. (2019). Peptide-based building blocks as structural elements for supramolecular Gd-containing MRI contrast agents. J. Pept. Sci..

[14] Preslar A.T., Parigi G., McClendon M.T., Sefick S.S., Moyer T.Y., Haney C.R., Waters E.A., MacRenaris K.W., Luchinat C., Stupp S.I. (2014). Gd(III)-Labeled Peptide Nanofibers for Reporting on Biomaterial Localization in Vivo. ACS Nano.

[15] Yin Y., Hu B., Yuan X., Cai L., Gao H., Yang Q. (2020). Nanogel: A Versatile Nano-Delivery System for Biomedical Applications. Pharmaceutics.

[16] Glangchai L.C., Caldorera-Moore M., Shi L., Roy K. (2008). Nanoimprint lithography based fabrication of shape-specific, enzymatically-triggered smart nanoparticles. J. Control. Release.

[17] Gheran C.V., Rigaux G., Callewaert M., Berquand A., Molinari M., Chuburu F., Voicu S.N., Dinischiotu A. (2018). Biocompatibility of Gd-loaded chitosan-hyaluronic acid nanogels as contrast agents for magnetic resonance cancer imaging. Nanomaterials.

[18] Carniato F., Tei L., Botta M., Ravera E., Fragai M., Parigi G., Luchinat C. (2020). ^1^H NMR Relaxometric Study of Chitosan-Based Nanogels Containing Mono- and Bis-Hydrated Gd(III) Chelates: Clues for MRI Probes of Improved Sensitivity. ACS Appl. Bio Mat..

[19] Carniato F., Ricci M., Tei L., Garello F., Terreno E., Ravera E., Parigi G., Luchinat C., Botta M. (2022). High Relaxivity with No Coordinated Waters: A Seemingly Paradoxical Behavior of [Gd(DOTP)]^5-^ Embedded in Nanogels. Inorg. Chem..

[20] Ischakov R., Adler-Abramovich L., Buzhansky L., Shekhter T., Gazit E. (2013). Peptide-based hydrogel nanoparticles as effective drug delivery agents. Bioorg. Med. Chem..

[21] Panda J.J., Kaul A., Kumar S., Alam S., Mishra A.K., Kundu G.C., Chauhan V.S. (2013). Modified dipeptide-based nanoparticles: Vehicles for targeted tumor drug delivery. Nanomedicine.

[22] Rosa E., Diaferia C., Gallo E., Morelli G., Accardo A. (2020). Stable Formulations of Peptide-Based Nanogels. Molecules.

[23] Tesauro D., Accardo A., Diaferia C., Milano V., Guillon J., Ronga L., Rossi F. (2019). Peptide-Based Drug-Delivery Systems in Biotechnological Applications: Recent Advances and Perspectives. Molecules.

[24] Diaferia C., Rosa E., Gallo E., Smaldone G., Stornaiuolo M., Morelli G., Accardo A. (2021). Supporting Hydrogels Based on Fmoc-Derivatized Cationic Hexapeptides for Potential Biomedical Applications. Biomedicines.

[25] Loo Y., Lakshmanan A., Ni M., Toh L.L., Wang S., Hauser C.A.E. (2015). Peptide Bioink: Self-Assembling Nanofibrous Scaffolds for Three-Dimensional Organotypic Cultures. Nano Lett..

[26] Lalli D., Carniato F., Tei L., Platas-Iglesias C., Botta M. (2021). Surprising Complexity of the [Gd(AAZTA)(H_2_O)_2_]^-^ Chelate Revealed by NMR in the Frequency and Time Domains. Inorg. Chem..

[27] Aime S., Calabi L., Cavallotti C., Gianolio E., Giovenzana G.B., Losi P., Maiocchi A., Palmisano G., Sisti M. (2004). [Gd-AAZTA]^-^: A New Structural Entry for an Improved Generation of MRI Contrast Agents. Inorg. Chem..

[28] Uggeri F., Aime S., Anelli P.L., Botta M., Brocchetta M., de Haen C., Ermondi G., Grandi M., Paoli P. (1995). Novel Contrast Agents for Magnetic Resonance Imaging. Synthesis and Characterization of the Ligand BOPTA and its Ln(III) Complexes (Ln = Gd, La, Lu). X-ray Structure of Disodium (TPS-9-145337286-C-S)-[4-Carboxy-5,8,ll-tris(carboxymethyI)-l-phenyl-2-oxa5,8,ll-triazatridecan-13-oato(5-)]gadolinate(2-) in a Mixture with its Enantiomer. Inorg. Chem..

[29] Xiao Y.D., Paudel R., Liu J., Ma C., Zhang Z.S., Zhou S.K. (2016). MRI contrast agents: Classification and application (Review). Int. J. Mol. Med..

[30] Aime S., Botta M., Terreno E. (2005). Gd(III)-based contrast agents for MRI. Adv. Inorg. Chem..

[31] Helm L., Morrow J.R., Bond C.J., Carniato F., Botta M., Braun M., Baranyai Z., Pujales-Paradela R., Regueiro-Figueroa M., Esteban-Gómez D. (2018). Contrast Agents for MRI: Experimental Methods.

[32] Caravan P., Ellison J.J., McMurry T.J., Lauffer R.B. (1999). Gadolinium(III) Chelates as MRI Contrast Agents: Structure, Dynamics, and Applications. Chem. Rev..

[33] Iyer A., Roeters S.J., Kogan V., Woutersen S., Claessens M.M.A.E., Subramaniam V. (2017). C-Terminal Truncated α-Synuclein Fibrils Contain Strongly Twisted β-Sheets. J. Am. Chem. Soc..

[34] Chronopoulou L., Margheritelli S., Toumia Y., Paradossi G., Bordi F., Sennato S., Palocci C. (2015). Biosynthesis and Characterization of Cross-Linked Fmoc Peptide-Based Hydrogels for Drug Delivery Applications. Gels.

