# Supplementary material for: Peptide-Based Hydrogels and Nanogels Containing Gd(III) Complexes as T1 Relaxation Agents
Source: Pharmaceuticals (Basel). 2022 Dec 16;15(12):1572. doi: 10.3390/ph15121572 (PMC9787396; doi:10.3390/ph15121572)
Supplement: Supplementary file 1 [file pharmaceuticals-15-01572-s001.zip › pharmaceuticals-2064170-supplementary.pdf]

Supporting Information

Peptide-based hydrogels and nanogels containing Gd(III) complexes as T_1 relaxation agents

Elisabetta Rosa, [1] Fabio Carniato,[2] Lorenzo Tei,[2] Carlo Diaferia,[1] Giancarlo Morelli,[1] Mauro Botta[2], Antonella Accardo[1]

[1]Department of Pharmacy, Research Centre on Bioactive Peptides (CIRPeB), University of Naples "Federico II", Via Montesano 49, 80131 Naples (Italy)

[2] Department of Science and Technological Innovation, University of Piemonte Orientale "A. Avogadro", Viale T. Michel 11, 15121 Alessandria (Italy)

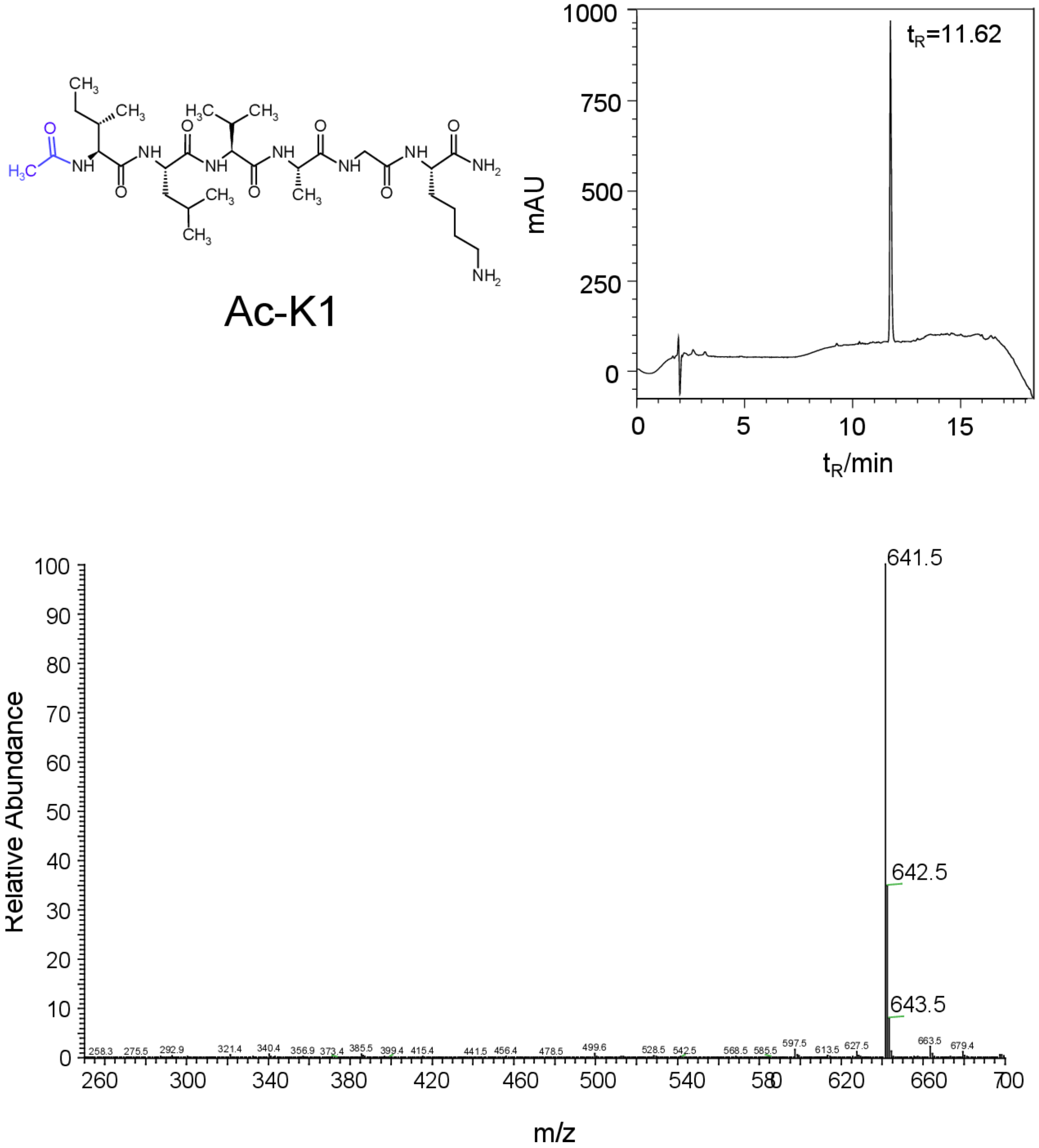

Figure S1: Chemical structure of Ac-K1 peptide with the corresponding RP-HPLC chromatogram and ESI mass spectrum

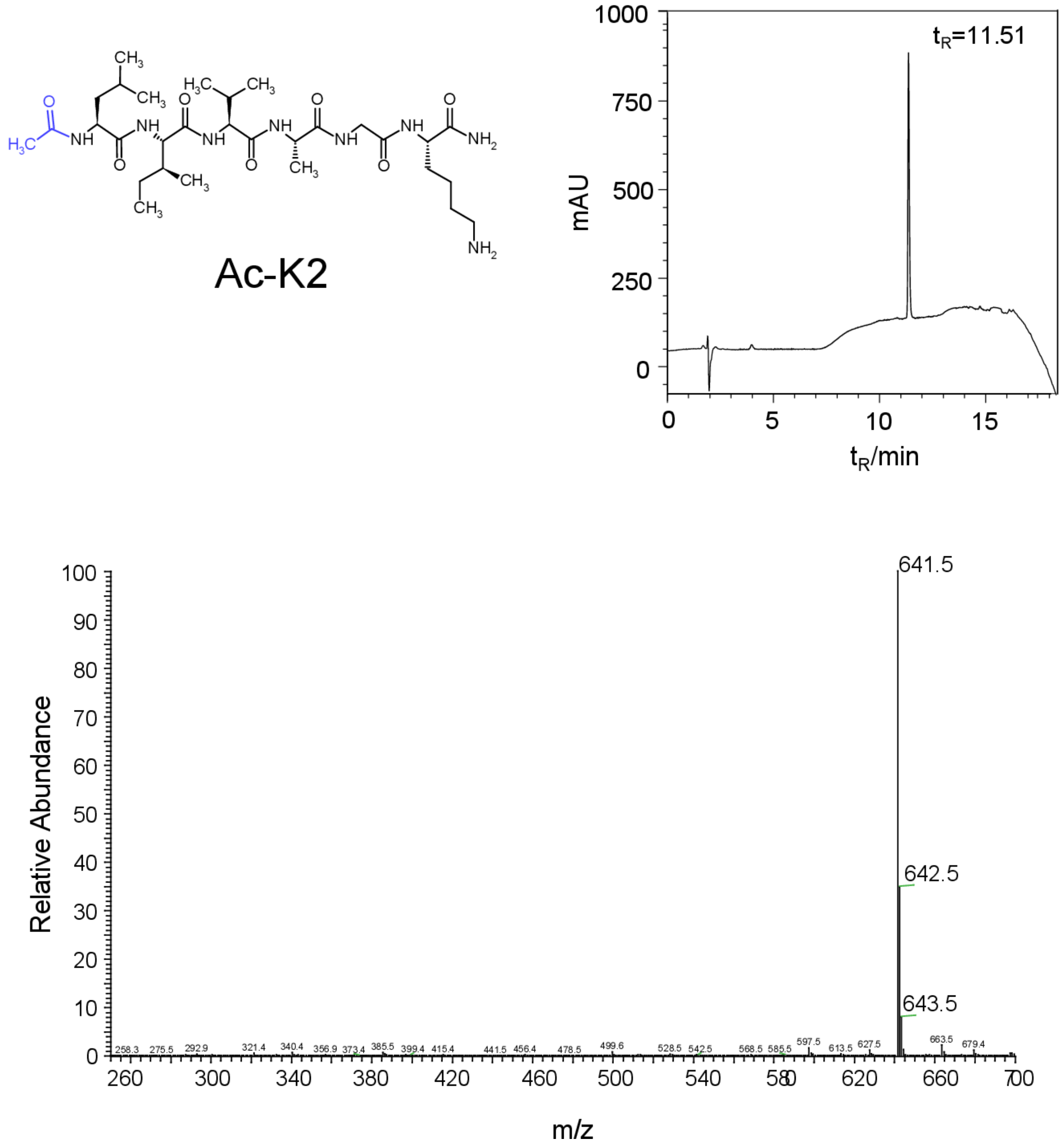

Figure S2: Chemical structure of Ac-K2 peptide with the corresponding RP-HPLC chromatogram and ESI mass spectrum

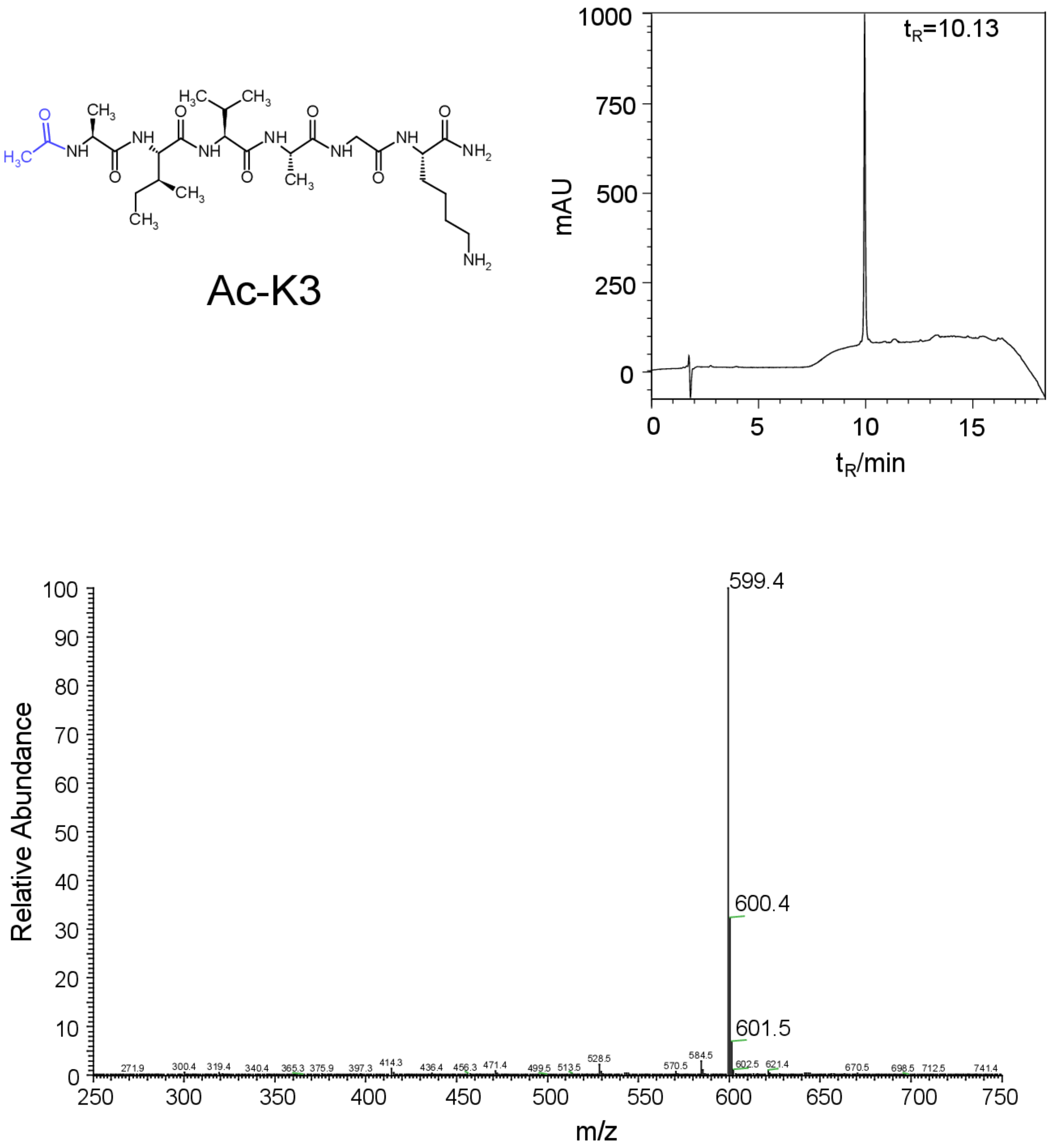

Figure S3: Chemical structure of Ac-K3 peptide with the corresponding RP-HPLC chromatogram and ESI mass spectrum

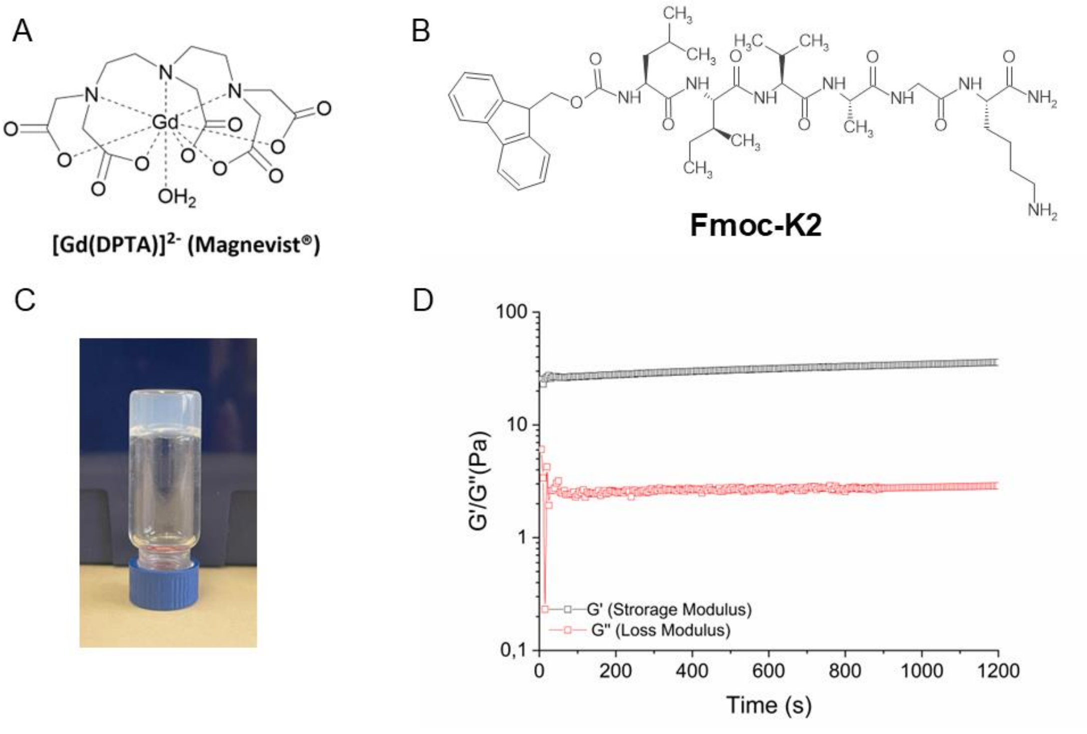

Figure S4: Schematic representation of $[Gd(DTPA)]^{2-}$ complex (A) and of Fmoc-K2 peptide (B). C) Inverted tube test for $[Gd(DTPA)]^{2-}$ filled Fmoc-K2 hydrogel. D) Time sweep rheological analysis of Fmoc-K2 hydrogel loaded with $[Gd(DTPA)]^{2-}$ reported as storage modulus (G') and loss modulus (G'').

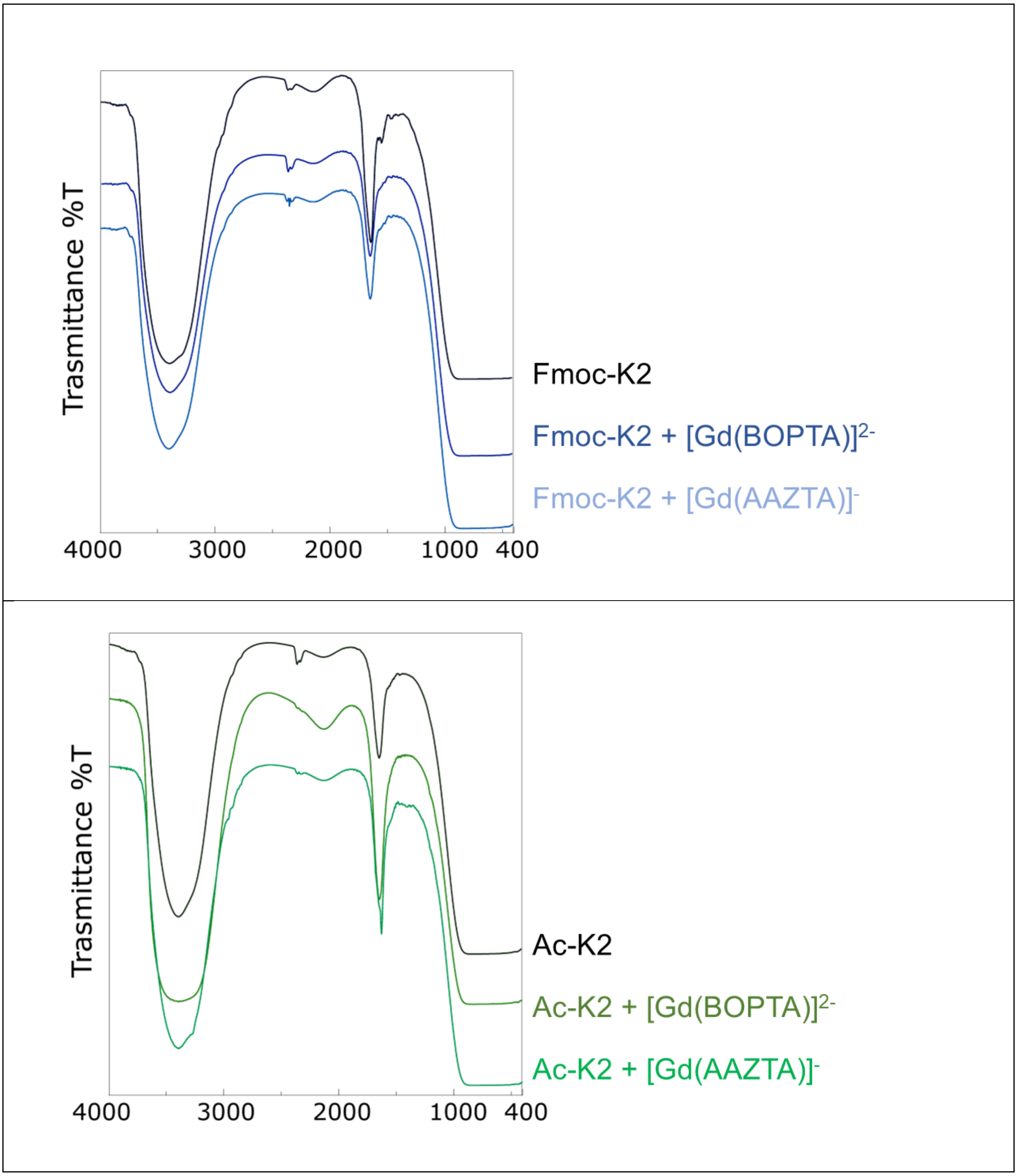

Figure S5: FTIR spectra of Fmoc-K2 and Ac-K2 empty hydrogels compared with hydrogels loaded with $[Gd(BOPTA)]^{2-}$ or $[Gd(AAZTA)]^{-}$ complexes.

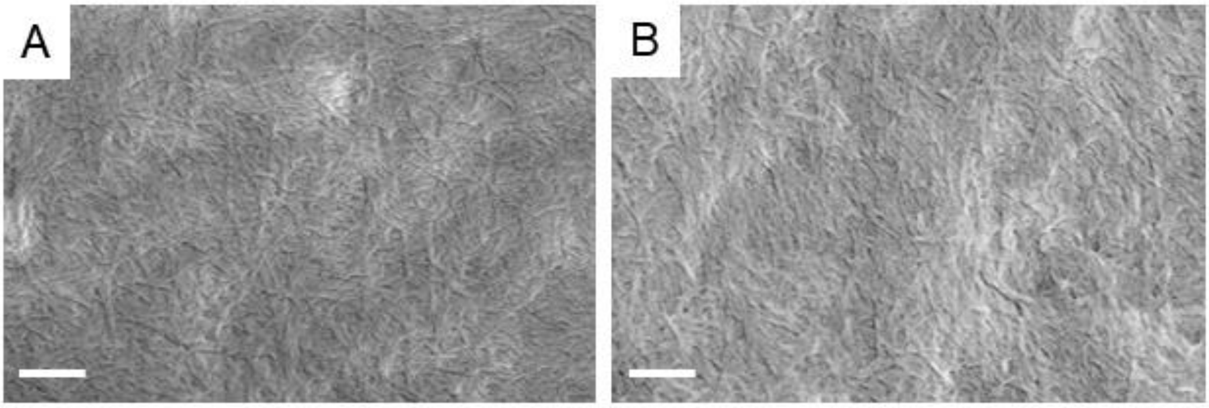

Figure S6: SEM micro-photos of Fmoc-K2 (A) and Fmoc-K3 (B) loaded with $[Gd(AAZTA)]^-$ (scale bar is 500 nm).

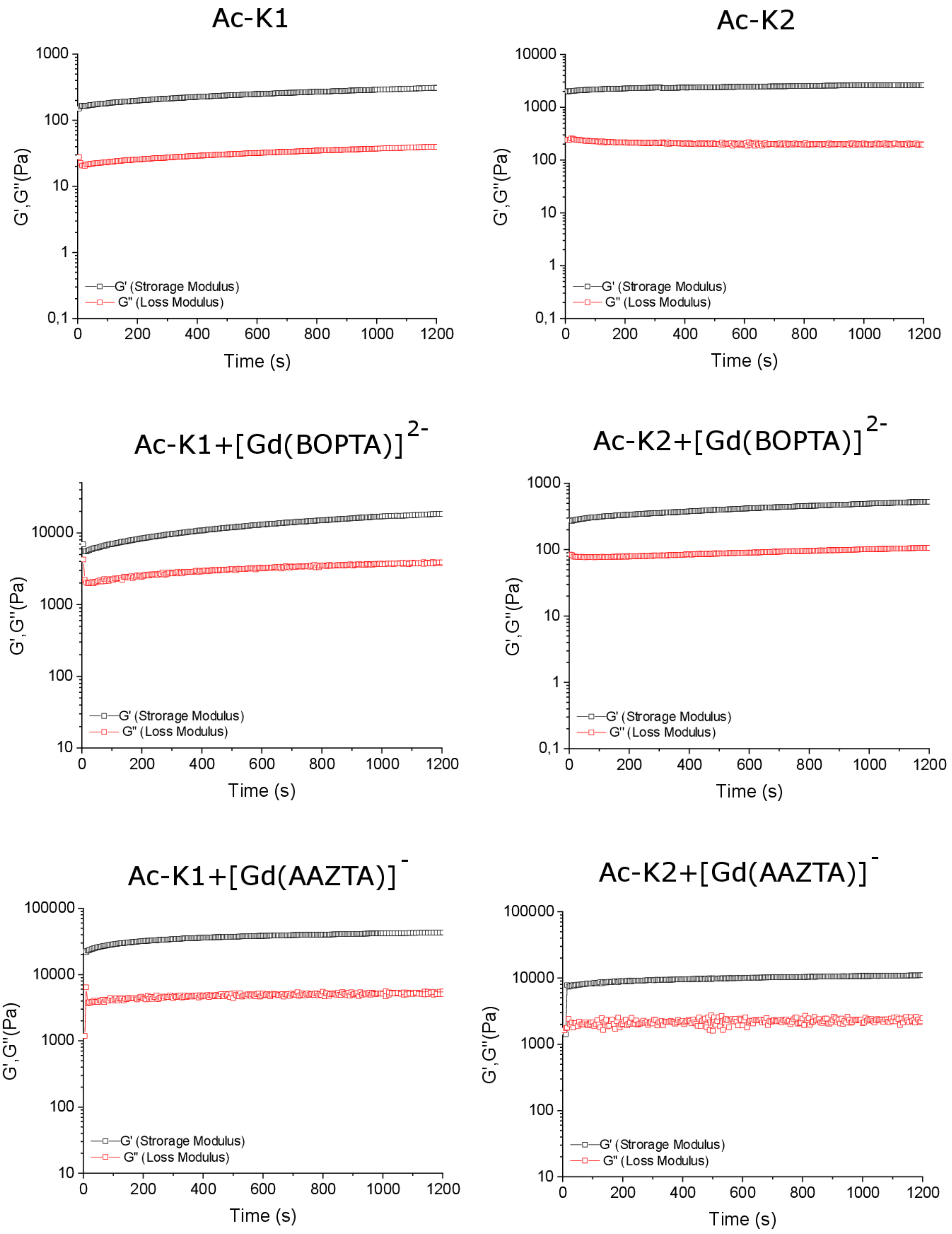

Figure S7: Rheological profiles of time sweep measurements for Ac-peptide series. G’ and G’’ are graphed in black and red, respectively.

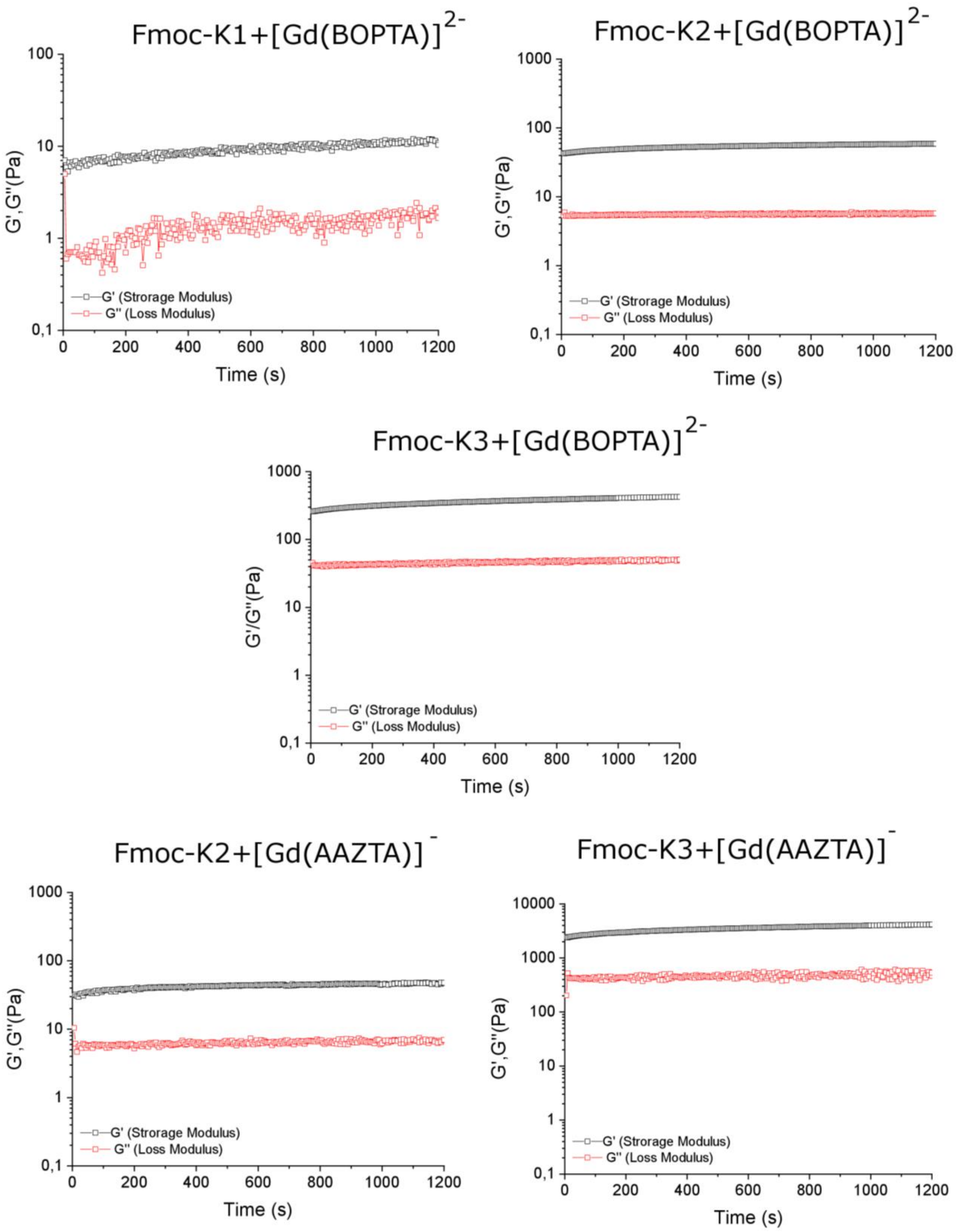

Figure S8: Rheological profiles of time sweep measurements for Fmoc-peptide series. G’ and G’’ are graphed in black and red, respectively.

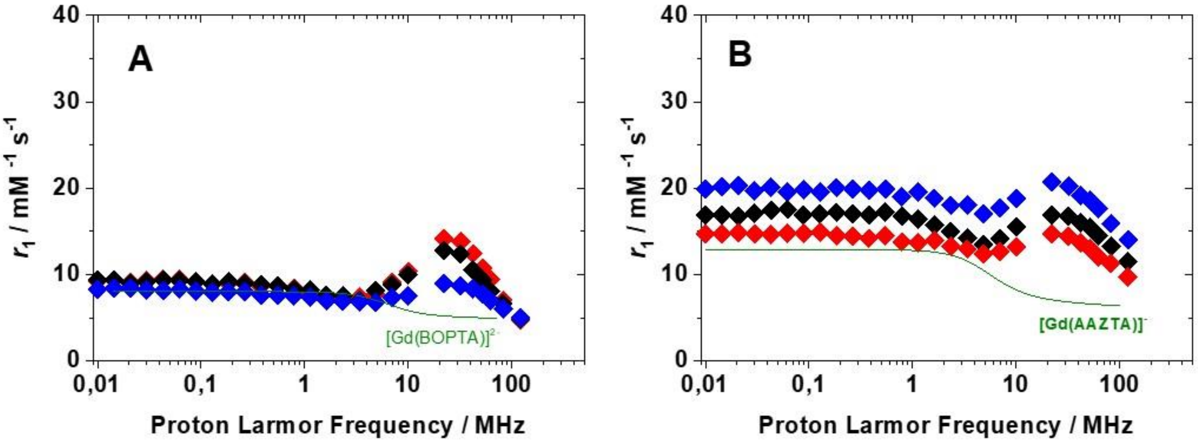

Figure S9: ^{1}H NMRD profiles of Fmoc-K3+[Gd(BOPTA)]$^{2-}$ (A), Fmoc-K3+[Gd(AAZTA)]$^{-}$ (B) at 283 (blue), 298 (black) and 310 K (red). The profiles of the corresponding chelates in aqueous solution are also reported as solid lines.

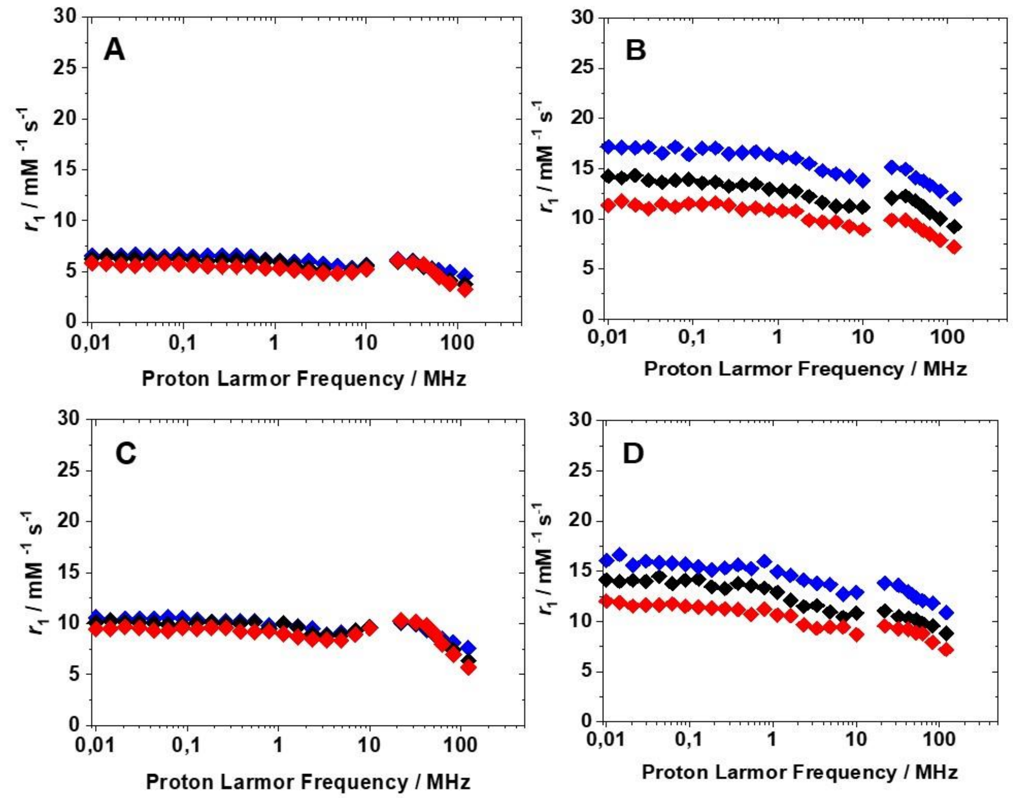

Figure S10: ^{1}H NMRD profiles of Ac-K1+[Gd(BOPTA)]$^{2-}$ (A), Ac-K1+[Gd(AAZTA)]$^-$ (B), Ac-K2+[Gd(BOPTA)]$^{2-}$ (C), Ac-K2+[Gd(AAZTA)]$^-$ (D) at 283 (blue), 298 (black) and 310 K (red).
